# Supplementary material for: Bivalent chromatin as a therapeutic target in cancer: An in silico predictive approach for combining epigenetic drugs
Source: PLoS Comput Biol. 2021 Jun 21;17(6):e1008408. doi: 10.1371/journal.pcbi.1008408 (PMC8248646; doi:10.1371/journal.pcbi.1008408)
Supplement: S1 Text — The file contains a more detailed description of the model formulation and model analysis, including a full derivation of the asymptotic model reduction. (PDF) [file pcbi.1008408.s001.pdf]

Bivalent chromatin as a therapeutic target in cancer: An *in silico* predictive approach for combining epigenetic drugs
Supplementary information

Tomás Alarcón[1,2,3*] Josep Sardanyés[2], Antoni Guillamon[4,2,5], Javier A. Menendez[6,7],

1 ICREA, Pg. Lluís Companys 23, 08010 Barcelona, Spain.
2 Centre de Recerca Matemàtica, Edifici C, Campus de Bellaterra, 08193 Cerdanyola del Vallès (Barcelona), Spain.
3 Departament de Matemàtiques, Universitat Autònoma de Barcelona, 08193 Cerdanyola del Vallès (Barcelona), Spain.
4 Departament de Matemàtiques, Universitat Politècnica de Catalunya, Av. Gregorio Marañón 44-50, 08028 Barcelona, Spain.
5 Institut de Matemàtiques de la UPC-BarcelonaTech (IMTech), Universitat Politècnica de Catalunya, Pau Gargallo 14, 08028 Barcelona, Spain.
6 Program Against Cancer Therapeutic Resistance (ProCURE), Metabolism and Cancer Group, Catalan Institute of Oncology, 17007 Girona, Spain.
7 Girona Biomedical Research Institute, 17190 Salt, Girona, Spain.

CONTENTS

S.1 Supplementary materials and methods **1**
S.1.1 Model formulation . . . 1
S.1.2 Asymptotic model reduction and singular perturbation analysis . . . 4
S.1.2.1 Asymptotic reduction of the stochastic model . . . 5
S.1.2.2 Asymptotic analysis of the forward equation of the reduced stochastic model for monomeric TF . . . 8
S.1.3 Deterministic model: Basins of attraction and bistability . . . 10
S.1.4 Parameter sensitivity analysis: Ensemble approach . . . 12
S.1.4.1 Ensemble description . . . 12
S.1.4.2 Classification into sub-ensembles . . . 12
S.1.5 Ensemble averages . . . 15

S.2 Supplementary Tables and Figures **16**

S.1 SUPPLEMENTARY MATERIALS AND METHODS

S.1.1 Model formulation

Variables. The variables considered in our model are given by:

- S_A: unmarked activating histone residues (H3K4).
- S_I: unmarked repressing histone residues (H3K27).
- E_A histone-modifying enzymes (histone methyltransferases, HMTs) that add marks (methylate) to H3K4 (MLL2).
- E_I: histone-modifying enzymes (HMTs) that add marks (methylate) to H3K27 (EZH2).
- E_{P_A} histone-modifying enzymes (histone demethylases, HDMs) that remove methylation marks from H3K4me3.
- E_{P_I}: histone-modifying enzymes (HDMs) that remove methylation marks from H3K27me3.
- C_A: complex that results from the methylation of the residue S_A by means of the enzyme E_A.
- C_I: complex that results from the methylation of the residue S_I by means of the enzyme E_I.
- P_A: marked activating substrates (trimethylated H3K4, H3K4me3).
- P_I: marked repressing substrates (trimethylated H3K27, H3K27me3).

- C_{P_A} : complex that results from the demethylation of the residue P_A by means of the enzyme E_{P_A}.
- C_{P_I} : complex that results from the demethylation of the residue P_I by means of the enzyme E_{P_I}.
- X: Transcription Factor (TF).
- B: bound sites within promoter.
- F: free binding sites within promoter.

Dynamics. The epigenetic regulation (ER) model consists of four enzymatic reactions, which we model by means of the Michaelis-Menten (MM) model of enzymatic catalysis (1, 2). The first reaction consists of the addition of a tri-methyl modification (me3) to the unmarked lysine residue H3K4 (S_A) by means of the corresponding enzyme (E_A which corresponds to MLL). The final product of such reaction is the trimethylated residue H3K4me3, which we denote as P_A:

$$S_A + E_A \underset{k_2(P_A,X)}{\overset{k_1(P_A,X)}{\rightleftharpoons}} C_A \xrightarrow{k_3(P_A,X)} P_A + E_A.$$

The second reaction is the enzyme catalysed removal of me3 from a H3K4me3 residue. In this case the enzyme responsible for such reaction is referred to as E_{P_A} and corresponds to the histone demethylase KDM:

$$P_A + E_{P_A} \underset{k_5}{\overset{k_4}{\rightleftharpoons}} C_{P_A} \xrightarrow{k_6} S_A + E_{P_A}.$$

The third reaction consists of the addition of a tri-methyl modification (me3) to the unmarked lysine residue H3K27 (S_I) mediated by the action of the associated enzyme (EZH2 which we denote as E_I). The final product of such reaction is the trimethylated residue H3K27me3, which we refer to as P_I:

$$S_I + E_I \underset{k_8(P_I,X)}{\overset{k_7(P_I)}{\rightleftharpoons}} C_I \xrightarrow{k_9(P_I)} P_I + E_I.$$

Finally, the last reaction is the enzyme-catalysed removal of me3 from a H3K27me3 residue. The KDM enzyme responsible for catalysing such reaction is referred to as E_{P_I}:

$$P_I + E_{P_I} \underset{k_{11}}{\overset{k_{10}}{\rightleftharpoons}} C_{P_I} \xrightarrow{k_{12}} S_I + E_{P_I}.$$

Note that some of the reaction rates are not constant. They are considered to be functions of P_A, P_I and the number of TF molecules, X, since the MM reactions must be supplemented with key feedback mechanisms (3–5). Such feedbacks are originated by the recruitment of epigenetic enzymes to unmodified sites by both epigenetic marks (6) and TFs (4), and have been previously modelled as regulating the reaction rates of the Michaelis-Menten reactions, k_i (4, 5, 7, 8). Regarding the coupling with the TF dynamics (see below), we consider a model whereby TFs enhances the recruitment of HME to H4K4me3 (i.e. activating substrates), $(k_1 = \bar{k}_1 X, k_2 = \bar{k}_2 X, k_3 = \bar{k}_3 X$, whilst hindering the recruitment of HMEs to H3K27me3, $(k_7 = \bar{k}_7, k_8 = \bar{k}_8 X, k_9 = \bar{k}_9)$ (4, 9, 10).

Furthermore, we consider recruitment of HMEs by modified nucleosomes. Modified nucleosomes create positive feedbacks so that a particular mark favours the recruitment of enzymes that promote further addition of that mark. This positive feedback phenomenon is referred to as *recruited histone modification*. Concerning our model, we consider that methylated nucleosomes recruit HMTs, which implies that the kinetic rates associated with HME_A and HME_I activity are proportional to the number of methylated nucleosomes (5, 7, 8). Depending on the model of TF-induced recruitment of HME, we have $(k_1 = a_1 + \hat{k}_1 P_A X, k_2 = a_2 + \hat{k}_2 P_A X, k_3 = a_3 + \hat{k}_3 P_A X)$ and $(k_7 = a_7 + \hat{k}_7 P_I, k_8 = a_8 + \hat{k}_8 P_I X, k_9 = a_9 + \hat{k}_9 P_I)$.

The gene regulation (GR) model is a simple variation of the Hill regulation model (2), and it accounts for the synthesis and degradation of TF molecules, X. We consider a basal (unregulated) rate of synthesis R, and a regulated synthesis mechanism. Such mechanism involves the binding of an α-mer of TF molecules which binds to the free binding sites of the promoter region of the gene (F). The rate of synthesis is assumed to be proportional to the number of bound promoter sites, B. Furthermore, we consider degradation of the TF molecule at a constant rate c_2:

$$\varnothing \overset{R}{\longrightarrow} X, F + \alpha X \underset{c_4(P_I)}{\overset{c_3(P_A)}{\rightleftharpoons}} B \overset{c_1}{\longrightarrow} X \overset{c_2}{\longrightarrow} \varnothing.$$

The so-called Hill parameter, α, is an integer which accounts for the cooperativity of the TF ($\alpha = 1$ if the TF is monomeric, $\alpha = 2$ if the TF is dimeric, and so on). The coupling with the ER model is through the dependence of the rates c_3 and c_4 on P_A and P_I: $c_3(P_A) = \hat{c}_3 P_A$ and $c_4(P_I) = \hat{c}_4 P_I$.

Conservation laws. The above model satisfies a number of conservation laws:

$$S_A + C_A + P_A + C_{P_A} = S_{A,0},\ S_I + C_I + P_I + C_{P_I} = S_{I,0},$$
$$E_A + C_A = E_{A,0},\ E_{P_A} + C_{P_A} = E_{P_A,0},\ E_I + C_I = E_{I,0},\ E_{P_I} + C_{P_I} = E_{P_I,0},$$
$$F + B = b_0.$$

From now on, we will substitute F by $b_0 - B$. The other conservation laws will be duly applied in further reductions. Therefore, we are left with 14 variables, which we conveniently order from slow to fast and collect them in the state vector of the system $Y := (X, P_A, P_I, S_A, S_I, E_A, E_{P_A}, E_I, E_{P_I}, C_A, C_{P_A}, C_I, C_{P_I}, B)$. In terms of these variables, the above reactions define 16 transition rates that are summarised in Table A.

Table A: Transition rates associated with the stochastic dynamics of the studied gene regulatory circuit. The vector of rates $r_i = (r_{i,j}) = (\Delta_i X, \Delta_i P_A, \Delta_i P_I, \Delta_i S_A, \Delta_i S_I, \Delta_i E_A, \Delta_i E_{P_A}, \Delta_i E_I, \Delta_i E_{P_I}, \Delta_i C_A, \Delta_i C_{P_A}, \Delta_i C_I, \Delta_i C_{P_I}, \Delta_i B)$ defines the stoichiometry associated with reaction i. The entries of the vector r_i are equal to zero unless otherwise stated in this table. For simplicity, we have dropped the hats. Since we are considering monomeric TF, $\alpha = 1$.

Transition rate	Event
$W_1(Y) = R + c_1 B$	$r_{1,1} = \Delta_1 X = +1$
$W_2(Y) = c_2 X$	$r_{2,1} = \Delta_2 X = -1$
$W_3(Y) = c_3\,(b_0 - B)\,P_A X$	$r_{3,1} = \Delta_3 X = -1$, $r_{3,14} = \Delta_3 B = +1$
$W_4(Y) = c_4 P_I B$	$r_{4,1} = \Delta_4 X = +1$, $r_{4,14} = \Delta_4 B = -1$
$W_5(Y) = (a_1 + k_1 P_A X) E_A S_A$	$r_{5,4} = \Delta_5 S_A = -1$, $r_{5,6} = \Delta_5 E_A = -1$, $r_{5,10} = \Delta_5 C_A = +1$
$W_6(Y) = (a_2 + k_2 P_A X) C_A$	$r_{6,4} = \Delta_6 S_A = +1$, $r_{6,6} = \Delta_6 E_A = +1$, $r_{6,10} = \Delta_6 C_A = -1$
$W_7(Y) = (a_3 + k_3 P_A X) C_A$	$r_{7,2} = \Delta_7 P_A = +1$, $r_{7,6} = \Delta_7 E_A = +1$, $r_{7,10} = \Delta_7 C_A = -1$
$W_8(Y) = k_4 P_A E_{P_A}$	$r_{8,2} = \Delta_8 P_A = -1$, $r_{8,7} = \Delta_8 E_{P_A} = -1$, $r_{8,11} = \Delta_8 C_{P_A} = +1$
$W_9(Y) = k_5 C_{P_A}$	$r_{9,2} = \Delta_9 P_A = +1$, $r_{9,7} = \Delta_9 E_{P_A} = +1$, $r_{9,11} = \Delta_9 C_{P_A} = -1$
$W_{10}(Y) = k_6 C_{P_A}$	$r_{10,4} = \Delta_{10} S_A = +1$, $r_{10,7} = \Delta_{10} E_{P_A} = +1$, $r_{10,11} = \Delta_{10} C_{P_A} = -1$
$W_{11}(Y) = (a_7 + k_7 P_I) E_I S_I$	$r_{11,5} = \Delta_{11} S_I = -1$, $r_{11,8} = \Delta_{11} E_I = -1$, $r_{11,12} = \Delta_{11} C_I = +1$
$W_{12}(Y) = (a_8 + k_8 P_I X) C_I$	$r_{12,5} = \Delta_{12} S_I = +1$, $r_{12,8} = \Delta_{12} E_I = +1$, $r_{12,12} = \Delta_{12} C_I = -1$
$W_{13}(Y) = (a_9 + k_9 P_I) C_I$	$r_{13,3} = \Delta_{13} P_I = +1$, $r_{13,8} = \Delta_{13} E_I = +1$, $r_{13,12} = \Delta_{13} C_I = -1$
$W_{14}(Y) = k_{10} P_I E_{P_I}$	$r_{14,3} = \Delta_{14} P_I = -1$, $r_{14,9} = \Delta_{14} E_{P_I} = -1$, $r_{14,13} = \Delta_{14} C_{P_I} = +1$
$W_{15}(Y) = k_{11} C_{P_I}$	$r_{15,3} = \Delta_{15} P_I = +1$, $r_{15,9} = \Delta_{15} E_{P_I} = +1$, $r_{15,13} = \Delta_{14} C_{P_I} = -1$
$W_{16}(Y) = k_{12} C_{P_I}$	$r_{16,5} = \Delta_{16} S_I = +1$, $r_{16,9} = \Delta_{16} E_{P_I} = +1$, $r_{16,13} = \Delta_{16} C_{P_I} = -1$

Scaling assumptions. An essential component of our model analysis involves the characteristic scales of the system. Let E_A, E_{P_A}, E_I, and E_{P_I} be the number of HME_A, HME_{P_A} HME_I, and HME_{P_I}, respectively, and X the number of TF molecules. We further define the following rescaled variables:

$$S_A = \Omega_S s_A, S_I = \Omega_S s_I, P_A = \Omega_S p_A, P_I = \Omega_S p_I,$$
$$E_A = \Omega_E \mathcal{E}_A, E_{P_A} = \Omega_E \mathcal{E}_{P_A}, E_I = \Omega_E \mathcal{E}_I, E_{P_I} = \Omega_E \mathcal{E}_{P_I},$$
$$C_A = \Omega_E c_A, C_{P_A} = \Omega_E c_{p_A}, C_I = \Omega_E c_I, C_{P_I} = \Omega_E c_{p_I},$$
$$X = \Omega_T x, B = \Omega_B b, F = \Omega_B f.$$

We further make the following assumptions regarding the characteristic scales, Ω_J:

$$\varepsilon_1 = \frac{\Omega_E}{\Omega_S} \ll 1, \varepsilon_2 = \frac{\Omega_S}{\Omega_T} \ll 1, \varepsilon_3 = \frac{\Omega_B}{\Omega_T} \ll 1$$

with $\varepsilon_3/\varepsilon_2 = \mathcal{O}(\varepsilon_1)$. The condition $\varepsilon_1 \ll 1$ is the usual Briggs-Haldane assumption leading to the Michaelis-Menten approximation for enzyme kinetics (1).

S.1.2 Asymptotic model reduction and singular perturbation analysis

We now proceed to formulate the stochastic model of the epigenetically-regulated gene circuit. The stochastic equations describing the dynamics of the model, encapsulated in the rates and stoichiometry described in Table A, are (11):

$$Y_j(t) = Y_j(0) + \sum_{i=1}^{16} r_{i,j} \mathscr{P}\left(\int_0^t W_i(Y(s))\,ds\right) \tag{S.1}$$

where $Y(t) = (Y_i(t))$, $i = 1, \ldots, 14$), is the state vector of the system, whose entries are the numbers of the different components of the system at time t (see Table A). $\mathscr{P}(\lambda)$ stands for a random number sampled from a Poisson distribution with parameter λ, i.e.

$$P(\mathscr{P}(\lambda) = n) = \frac{\lambda^n}{n!} e^{-\lambda}.$$

Each of the Poisson variables in Eq. (S.1) counts the number of times that reaction i has occurred in the time interval $(0,t]$. In other words, Eq. (S.1) represents the process as a Markovian jump process, where the state of the system, $Y(t)$, changes abruptly at discrete, random arrival times with exponentially distributed interarrival waiting times (11).

In order to make some analytical progress, we exploit the presence of multiple time scales in Eq. (S.1). Such a feature is ubiquitous in biological systems and has been previously used to formulate efficient numerical algorithms and asymptotic model reduction methods (12–24). In particular, under the scaling assumptions described in Section S.1.1, we have shown that multiple time scales are present in the stochastic dynamics, and proposed asymptotic model reductions methods to simplify the stochastic dynamics (8, 21, 23, 26).

In order to proceed with our asymptotic analysis, we consider the scaling of the state variables defined in Section S.1.1. We further rescale the reaction rates $W_i(Y)$ according to the method described in (23), obtaining:

$$W_i(Y) = k_1 \Omega_E \Omega_T \Omega_S^2 \omega_i(y) + \mathscr{O}(\Omega^0)$$

where

$$y = (y_i) := (x, p_A, p_I, s_A, s_I, e_A, e_{p_A}, e_I, e_{p_I}, c_A, c_{p_A}, c_I, c_{p_I}, b) \tag{S.2}$$

is the rescaled state vector. Further, by defining the rescaled time variable $\tau = k_1 \Omega_E \Omega_S^2 t$, Eq. (S.1) reads:

$$y_1(\tau) = y_1(0) + \sum_{i=1}^{16} r_{i,1} \frac{1}{\Omega_T} \mathscr{P}\left(\Omega_T \int_0^\tau \omega_i(y(s))\,ds\right), \tag{S.3}$$

$$y_j(\tau) = y_j(0) + \sum_{i=1}^{16} r_{i,j} \frac{1}{\Omega_S} \mathscr{P}\left(\Omega_S \frac{1}{\varepsilon_2} \int_0^\tau \omega_i(y(s))\,ds\right), \quad j = 2, \ldots, 5, \tag{S.4}$$

$$y_j(\tau) = y_j(0) + \sum_{i=1}^{16} r_{i,j} \frac{1}{\Omega_E} \mathscr{P}\left(\Omega_E \frac{1}{\varepsilon_1} \frac{1}{\varepsilon_2} \int_0^\tau \omega_i(y(s))\,ds\right), \quad j = 6, \ldots, 13, \tag{S.5}$$

$$y_{14}(\tau) = y_{14}(0) + \sum_{i=1}^{16} r_{i,14} \frac{1}{\Omega_B} \mathscr{P}\left(\Omega_B \frac{1}{\varepsilon_3} \int_0^\tau \omega_i(y(s))\,ds\right), \tag{S.6}$$

where we recall that $y_1 = x$ and $y_{14} = b$, see the state vector (S.2). From Eqs. (S.4)-(S.6) and the assumptions regarding the characteristic scales Ω_J (see Section S.1.1), it is straightforward to observe that the system exhibits separation of time scales. Under such scaling assumptions, we are able to distinguish three regimes:

1. A *fast dynamics* in which the HMEs and associated complexes reach quasi-equilibrium with the substrates, products, TF, and marks. Also, the binding sites in the promoter of the gene reach quasi-equilibrium with TFs and ER products. Such dynamics is associated with the so-called fast variables y_j, $j = 6, \ldots, 13$ and $y_{14} = b$, see Eqs. (S.7)-(S.8) in Section S.1.2.1.

2. An *intermediate dynamics* in which epigenetic substrates and products reach quasi-equilibrium with TF and marks. This regime corresponds to the dynamics of $y_2 = p_A$, $y_3 = p_I$, $y_4 = s_A$ and $y_5 = s_I$. Using conservation laws, this dynamics is reduced to system (S.17)-(S.18) in the variables p_A and p_I, which can be further simplified by a process of self-averaging that leads to equations (S.19)-(S.20).

3. A *slow dynamics* in which the TF, $y_1 = x$, finally reaches equilibrium. The *reduced stochastic model for monomeric self-activating TF*, see (S.21)-(S.23), describes the dynamics of the slow variable x together with the reduced intermediate dynamics in the variables p_A and p_I. The resulting three-dimensional system is the basis of the mean-field model that we analyse in Section S.1.3.

The asymptotic model reduction is carried out in three stages. In the first step, the *fast dynamics* is studied under adiabatic conditions with respect to the *intermediate* and *slow* variables, i.e. the latter variables are considered as frozen and the fast variables can be analysed, specifically regarding their QSSD, with the intermediate and slow variables set to constant. In the second stage, the intermediate variables are studied under adiabatic conditions with respect to the slow variables and by sampling the fast variables from their fast QSSD. The third stage consists of studying the slow variables with the fast and intermediate in quasi-equilibrium, i.e. sampled from their associated QSSD.

S.1.2.1 Asymptotic reduction of the stochastic model

We will now proceed to illustrate the details of the method of asymptotic model reduction based on multiple time scale analysis of the stochastic model.

Inner solution. We continue our analysis by performing a further rescaling of the time variable: $\mathscr{T} = \varepsilon_1^{-1}\varepsilon_2^{-1}\tau$. Upon applying this rescaling, it is straightforward to see that the transition rates affecting the slow variable, $y_1 = x$, becomes $\mathscr{O}(\varepsilon_1\varepsilon_2)$. Furthermore, the transition rates in the stochastic equations for $y_2(\mathscr{T})$, $y_3(\mathscr{T})$, $y_4(\mathscr{T})$, and $y_5(\mathscr{T})$ become $\mathscr{O}(\varepsilon_2)$. As a consequence we have that $y_1(T) = x \simeq$cnt., and also that $y_2(\mathscr{T}) = p_A \simeq$ cnt., $y_3(\mathscr{T}) = p_I \simeq$ cnt., $y_4(\mathscr{T}) = s_A \simeq$ cnt., and $y_5(\mathscr{T}) = s_I \simeq$ cnt. As a result of this rescaling, the rates of the the fast variables expressed as functions of $\mathscr{T}$, become $\mathscr{O}(1)$ so that:

$$y_j(\mathscr{T}) = y_j(0) + \sum_{i=1}^{16} r_{i,j}\frac{1}{\Omega_E}\mathscr{P}\left(\Omega_E\int_0^{\mathscr{T}}\omega_i(y(s))\,ds\right),\; j = 6,\ldots,13, \tag{S.7}$$

$$b(\mathscr{T}) = b(0) + \sum_{i=1}^{16} r_{i,14}\frac{1}{\Omega_B}\mathscr{P}\left(\Omega_B\varepsilon_1\frac{\varepsilon_2}{\varepsilon_3}\int_0^{\mathscr{T}}\omega_i(y(s))\,ds\right), \tag{S.8}$$

where $\varepsilon_1\frac{\varepsilon_2}{\varepsilon_3} = \mathscr{O}(1)$. The stochastic dynamics described by Eqs. (S.7)-(S.8) proceeds under (i.e. conditioned to) $y_2(\mathscr{T}) = p_A \simeq$ cnt., $y_3(\mathscr{T}) = p_I \simeq$ cnt., $y_4(\mathscr{T}) = s_A \simeq$ cnt., and $y_5(\mathscr{T}) = s_I \simeq$ cnt., and also $y_1(\mathscr{T}) = x \simeq$cnt.

Since the fast variables relax towards their quasi-equilibrium distribution, i.e. conditioned to the instantaneous value of the slower variables, we study the steady state behaviour of Eqs. (S.7)-(S.8). Such distributions have been previously studied in Ref. (8). In particular, the quasi-steady state distribution (QSSD) of the fast variable B is a binomial, whose probability generating function (PGF) is given by:

$$G_B(z) = \left(\frac{\xi_4 p_I + \xi_3 p_A x z}{\xi_4 p_I + \xi_3 p_A x}\right)^{b_0}. \tag{S.9}$$

Similarly, the QSSD of each of the four HMEs is also a binomial (see (8)). The PGF for the QSSD of HME_A and HME_{P_A} are given by:

$$G_1(z) = (1 - p_1(s_A,x) + p_1(s_A,x)z)^{E_{A,0}}, \tag{S.10}$$

$$G_2(z) = (1 - p_2(s_A,x) + p_2(s_A,x)z)^{E_{P_A,0}}. \tag{S.11}$$

The quantities $p_1(s_A,x)$ and $p_2(s_A,x)$ are defined as:

$$p_1(s_A,x) \equiv \frac{\alpha_2 + \kappa_2(p_A,x) + \alpha_3 + \kappa_3(p_A,x)}{(\alpha_1 + \kappa_1(p_A,x))s_A + \alpha_2 + \kappa_2(p_A,x) + \alpha_3 + \kappa_3(p_A,x)},$$

$$p_2(s_A,x) \equiv \frac{\kappa_5 + \kappa_6}{\kappa_4 p_A + \kappa_5 + \kappa_6}, \tag{124}$$

where κ_i stand for the rescaled versions of their counterparts, k_i (see Table S.1), with $\kappa_1(p_A,x) = \kappa_1 p_A x$, $\kappa_2(p_A,x) = \kappa_2 p_A x$, and $\kappa_2(p_A,x) = \kappa_3 p_A x$.

Furthermore, since (from the conservation laws) $s_A + p_A + \varepsilon_2(c_A + c_{PA}) = \frac{S_{A,0}}{\Omega_S} \equiv \rho_A$ and $\varepsilon_2 \ll 1$, we have that $p_A \simeq \rho_A - s_A$.

Regarding HME_I and HME_{P_I}, we have:

$$G_3(z) = ((1 - p_3(s_I, x)) + p_3(s_I, x)z)^{E_{I,0}}, \tag{S.12}$$

$$G_4(z) = ((1 - p_4(s_I, x)) + p_4(s_I, x)z)^{E_{P_I,0}}, \tag{S.13}$$

where

$$p_3(s_I, x) \equiv \frac{\alpha_8 + \kappa_8(p_I, x) + \alpha_9 + \kappa_9(p_I, x)}{(\alpha_7 + \kappa_7(p_I, x))s_I + \alpha_8 + \kappa_8(p_I, x) + \alpha_9 + \kappa_9(p_I, x)},$$

with $\kappa_7(p_I, x) = \kappa_7 p_I x$, $\kappa_8(p_I, x) = \kappa_8 p_I x$, and $\kappa_9(p_I, x) = \kappa_3 p_I x$ (see Table B), and

$$p_4(s_I, x) \equiv \frac{\kappa_{11} + \kappa_{12}}{\kappa_{10} p_I + \kappa_{11} + \kappa_{12}}.$$

In this case we also have that, since (from the conservation laws) $s_I + p_I + \varepsilon_2(c_I + c_{PI}) = \frac{S_{I,0}}{\Omega_S} \equiv \rho_I$ and $\varepsilon_2 \ll 1$, $p_I \simeq \rho_I - s_I$.

Table B: Parameter groupings, see Materials and Methods in the main manuscript. The parameter ρ is the rescaled version of R: $\rho = \frac{R}{k_1 \Omega_E \Omega_T \Omega_S^2}$.

Methylation/demethylation of H3K4
$A_1 = \frac{\alpha_1}{\kappa_1}$, $A_3 = \frac{\alpha_3}{\kappa_3}$, $B_2 = \frac{\alpha_2+\alpha_3}{\kappa_1}$, $K_2 = \frac{\kappa_2+\kappa_3}{\kappa_1}$, $K_5 = \frac{\kappa_5+\kappa_6}{\kappa_4}$
$K_6 = \frac{\kappa_6}{\kappa_5}$, $\Lambda_A = \kappa_3 \tilde{\rho}_A$, $\lambda_A = \frac{\kappa_5 \tilde{\rho}_I}{\kappa_3 \tilde{\rho}_A}$
Methylation/demethylation of H3K27
$A_7 = \frac{\alpha_7}{\kappa_7}$, $A_9 = \frac{\alpha_9}{\kappa_9}$, $B_8 = \frac{\alpha_8+\alpha_9}{\kappa_7}$, $K_8 = \frac{\kappa_8}{\kappa_7}$, $K_9 = \frac{\kappa_9}{\kappa_7}$, $K_{11} = \frac{\kappa_{11}+\kappa_{12}}{\kappa_{10}}$
$K_{12} = \frac{\kappa_{12}}{\kappa_{11}}$, $\Lambda_I = \kappa_9 \tilde{\rho}_{P_A}$, $\lambda_I = \frac{\kappa_{11} \tilde{\rho}_{P_I}}{\kappa_9 \tilde{\rho}_{P_A}}$
Gene regulatory network
$C_1 = \frac{\beta_0 \xi_1}{\rho}$, $C_4 = \frac{\beta_0 \xi_4}{\rho}$, $\lambda_x = \frac{\xi_4}{\xi_3}$

Intermediate solution. We continue the analyses of Eqs. (S.4)-(S.6) by rescaling the time variable so that $T = \varepsilon_2^{-1}\tau$. Again, it is straightforward to check that all the transition rates of the stochastic equations for the slow variable $y_1 = x$ becomes $\mathscr{O}(\varepsilon_2)$. This implies that, up to leading order, $y_1(T) = x$ remains approximately constant. The stochastic equations for the intermediate and fast variables become:

$$y_j(T) = y_j(0) + \sum_{i=1}^{16} r_{i,j} \frac{1}{\Omega_S} \mathscr{P}\left(\Omega_S \int_0^T \omega_i(y(s))\, ds\right), \quad j = 2, \ldots, 5, \tag{S.14}$$

$$y_j(T) = y_j(0) + \sum_{i=1}^{16} r_{i,j} \frac{1}{\Omega_E} \mathscr{P}\left(\Omega_E \frac{1}{\varepsilon_3} \int_0^T \omega_i(y(s))\, ds\right), \quad j = 6, \ldots, 13, \tag{S.15}$$

$$b(T) = b(0) + \sum_{i=1}^{16} r_{i,14} \frac{1}{\Omega_B} \mathscr{P}\left(\Omega_B \frac{\varepsilon_2}{\varepsilon_3} \int_0^T \omega_i(y(s))\, ds\right), \tag{S.16}$$

where $b(T) = y_{14}(T)$. The evolution described by Eqs. (S.14)-(S.16) occurs under $y_1(T) = x \simeq$cnt. Note that, by representing the dynamics as a function of the variable T, the rates of $y_2 = p_A$, $y_3 = p_I$, $y_4 = s_A$ and $y_5 = s_I$ become $\mathscr{O}(1)$. By contrast, $y_j(T)$, $j = 6, \ldots, 13$ and $b = y_{14}(T)$ remain as fast variables since their rates are $\mathscr{O}(\varepsilon_3^{-1})$ and $\mathscr{O}(\frac{\varepsilon_2}{\varepsilon_3}) = \mathscr{O}(\varepsilon_1^{-1})$, respectively. This implies that the latter will swiftly relax on to their QSSD, and therefore their values can be sampled from the QSSDs

derived in the previous section conditioned to the instantaneous values of the slow and intermediate variables. Therefore the dynamics in the intermediate regime is described by:

$$p_A(T) = p_A(0) + \sum_{i=1}^{16} r_{i,2} \frac{1}{\Omega_S} \mathscr{P}\left(\Omega_S \int_0^T \omega_i(y(s))\, ds\right), \tag{S.17}$$

$$p_I(T) = p_I(0) + \sum_{i=1}^{16} r_{i,3} \frac{1}{\Omega_S} \mathscr{P}\left(\Omega_S \int_0^T \omega_i(y(s))\, ds\right), \tag{S.18}$$

which proceeds under constant slow variable ($y_1(T) = x \simeq$cnt.), and with fast variables sampled from their QSSDs:

$$b = \Omega_B^{-1} \mathrm{Bin}(p_b(p_A, p_I, x), b_0),$$

$$E_A = \Omega_E^{-1} \mathrm{Bin}(p_1(s_A, x), E_{A,0}),\ c_A = \tilde{\rho}_A - E_A,$$

$$E_{P_A} = \Omega_E^{-1} \mathrm{Bin}(p_2(s_A, x), E_{P_A,0}),\ c_{p_A} = \tilde{\rho}_{p_A} - E_{P_A},$$

$$E_I = \Omega_E^{-1} \mathrm{Bin}(p_3(s_I, x), E_{I,0}),\ c_I = \tilde{\rho}_I - E_I,$$

$$E_{P_I} = \Omega_E^{-1} \mathrm{Bin}(p_4(s_I, x), E_{P_I,0}),\ c_{p_I} = \tilde{\rho}_{p_I} - E_{P_I},$$

where $\tilde{\rho}_J \equiv \frac{E_{J,0}}{\Omega_E}$, with $J \in \{A, P_A, I, P_I\}$, are defined from the conservation laws, and

$$p_b(p_A, p_I, x) = \frac{\xi_3 p_A x}{\xi_4 p_I + \xi_3 p_A x}.$$

We also use that $s_A \simeq \rho_A - p_A$ and $s_I \simeq \rho_I - p_I$. The fast variables thus become an additional random perturbation to the (stochastic) evolution equations for s_A and s_I.

An additional simplification of the dynamics within the intermediate regime is possible by taking a leaf from the book of Cao et al. (14, 15). According to Cao et al., when separation of time scales occur, the fast reactions fire up so so many times (within the characteristic time scale for variation of the slower ones) that a process of self-averaging occurs. In the present situation, this implies that we can approximate the fast variables by their averages over the corresponding QSSD in Eqs. (S.17)-(S.18). Under this assumption, Eqs. (S.17)-(S.18) can be written as:

$$p_A(T) = p_A(0) + \frac{1}{\Omega_S} \mathscr{P}\left(\Omega_S \int_0^T B_A(p_A(s), x) ds\right) - \frac{1}{\Omega_S} \mathscr{P}\left(\Omega_S \int_0^T D_A(p_A(s), x) ds\right), \tag{S.19}$$

$$p_I(T) = p_I(0) + \frac{1}{\Omega_S} \mathscr{P}\left(\Omega_S \int_0^T B_I(p_I(s), x) ds\right) - \frac{1}{\Omega_S} \mathscr{P}\left(\Omega_S \int_0^T D_I(p_I(s), x) ds\right), \tag{S.20}$$

where (see Table B):

$$B_A(p_A, x) = \Lambda_A \frac{(\rho_A - p_A)(A_1 + p_A x)(A_3 + p_A x)}{(\rho_A - p_A)(A_1 + p_A x) + B_2 + K_2 p_A x},$$

$$D_A(p_A, x) = \Lambda_A \lambda_A \frac{K_6 p_A}{p_A + K_5},$$

$$B_I(p_I, x) = \Lambda_I \frac{(\rho_I - p_I)(A_7 + p_I x)(A_9 + p_I x)}{(\rho_I - p_I)(A_7 + p_I x) + B_8 + (K_8 x + K_9 x) p_I},$$

and

$$D_I(p_I, x) = \Lambda_I \lambda_I \frac{K_{12} p_I}{p_I + K_{11}}.$$

Eqs. (S.19)-(S.20) define two (independent) nonlinear birth-and-death processes for the evolution of P_A and P_I.

Reduced stochastic model for monomeric self-activating TF. By taking into account the results of the previous two sections, and by expressing the dynamics in terms of the time variable τ, we can finally write the reduced stochastic system. The reduced stochastic model reads:

$$x(\tau) = x(0) + \frac{1}{\Omega_T}\mathscr{P}\left(\Omega_T\int_0^\tau B_x(x,p_A,p_I)ds\right) - \frac{1}{\Omega_T}\mathscr{P}\left(\Omega_T\int_0^\tau D_x(x,p_A,p_I)ds\right), \tag{S.21}$$

$$p_A(\tau) = p_A(0) + \frac{1}{\Omega_S}\mathscr{P}\left(\Omega_S\frac{1}{\varepsilon_2}\int_0^\tau B_A(p_A(s),x)ds\right) - \frac{1}{\Omega_S}\mathscr{P}\left(\Omega_S\frac{1}{\varepsilon_2}\int_0^\tau D_A(p_A(s),x)ds\right), \tag{S.22}$$

$$p_I(\tau) = p_I(0) + \frac{1}{\Omega_S}\mathscr{P}\left(\Omega_S\frac{1}{\varepsilon_2}\int_0^\tau B_I(p_I(s),x)ds\right) - \frac{1}{\Omega_S}\mathscr{P}\left(\Omega_S\frac{1}{\varepsilon_2}\int_0^\tau D_I(p_I(s),x)ds\right), \tag{S.23}$$

where

$$B_x(x,p_A,p_I) = \rho\left[1 + (C_1 + C_4 p_I)\frac{p_A x}{\lambda_x p_I + p_A x}\right],$$

$$D_x(x,p_A,p_I) = \rho\left[C_2 x + C_4 p_I\frac{p_A x}{\lambda_x p_I + p_A x}\right].$$

The mean-field model equations (see Eqs. (1)-(3) of main text) can be now derived, by considering the limit $\Omega_T \to \infty$ and $\Omega_S \to \infty$. Defining

$$q_J = \lim_{\Omega_S \to +\infty} p_J, \quad J \in \{A, I\},$$

and imposing that the ratio $\Omega_S/\Omega_T = \varepsilon_2$ remains constant, we can use the law of large numbers for Poisson random variables. Notice that, for simplicity, we keep the notation x for the TF variable. Informally stated such law reads $\frac{1}{\Omega}\mathscr{P}(\Omega\varphi) \to \varphi$ when $\Omega \to \infty$ (see reference (25) for a rigorous statement of this result). By applying this limit to the system (S.21)-(S.23) we obtain

$$x(\tau) = x(0) + \int_0^\tau B_x(x,q_A,q_I)ds - \int_0^\tau D_x(x,q_A,q_I)ds,, \tag{S.24}$$

$$q_A(\tau) = q_A(0) + \frac{1}{\varepsilon_2}\int_0^\tau B_A(q_A(s),x)ds - \frac{1}{\varepsilon_2}\int_0^\tau D_A(q_A(s),x)ds, \tag{S.25}$$

$$q_I(\tau) = q_I(0) + \frac{1}{\varepsilon_2}\int_0^\tau B_I(q_I(s),x)ds - \frac{1}{\varepsilon_2}\int_0^\tau D_I(q_I(s),x)ds. \tag{S.26}$$

Finally, by taking the derivative with respect to τ, the mean-field deterministic model Eqs. (1)-(3) in the main text are obtained.

S.1.2.2 Asymptotic analysis of the forward equation of the reduced stochastic model for monomeric TF

The forward (Master) equation is the equation satified by the probability density function $\Phi(x, p_A, p_I, \tau)$, i.e. the probability density of the system (S.21)-(S.23) to be in state (x, p_A, p_I) at time τ, is given by (27):

$$\frac{\partial\Phi}{\partial\tau} = \mathscr{H}_x(x,\partial_x)\Phi + \frac{1}{\varepsilon_2}\left(\mathscr{H}_A(p_A,\partial_{p_A}) + \mathscr{H}_I(p_I,\partial_{p_I})\right)\Phi \tag{S.27}$$

where the operators $\mathscr{H}_x$, $\mathscr{H}_A$, and $\mathscr{H}_I$ are given by:

$$\mathscr{H}_x\Phi = \Omega_T\left(\left(e^{\partial_x} - 1\right)B_x(x,p_A,p_I)\Phi(x,p_A,p_I,\tau) + \left(e^{-\partial_x} - 1\right)D_x(x,p_A,p_I)\Phi(x,p_A,p_I,\tau)\right),$$

$$\mathscr{H}_A\Phi = \Omega_S\left(\left(e^{\partial_{p_A}} - 1\right)B_A(x,p_A,p_I)\Phi(x,p_A,p_I,\tau) + \left(e^{-\partial_{p_A}} - 1\right)D_A(x,p_A,p_I)\Phi(x,p_A,p_I,\tau)\right),$$

$$\mathscr{H}_I\Phi = \Omega_S\left(\left(e^{\partial_{p_I}} - 1\right)B_I(x,p_A,p_I)\Phi(x,p_A,p_I,\tau) + \left(e^{-\partial_{p_I}} - 1\right)D_I(x,p_A,p_I)\Phi(x,p_A,p_I,\tau)\right),$$

and the operator $e^{r\partial x}$ is defined as:

$$e^{r\partial x} = \sum_{n=0}^{\infty}\frac{r^n}{n!}\frac{\partial^n}{\partial x^n},$$

so that $e^{r\partial x}f(x) = f(x+r)$, provided f is C^∞.

In order to proceed forward we propose that the PDF $\Phi(x, p_A, p_I, \tau)$ can be expanded in powers of ε_2: $\Phi = \Phi_0 + \varepsilon_2 \Phi_1 + \mathscr{O}(\varepsilon_2^2)$ (28, 29). By introducing such an expansion in Eq. (S.27), it is straightforward to find that Φ_0 and Φ_1 satisfy:

$$(\mathscr{H}_A(p_A, \partial_{p_A}) + \mathscr{H}_I(p_I, \partial_{p_I}))\,\Phi_0 = 0, \tag{S.28}$$

$$(\mathscr{H}_A(p_A, \partial_{p_A}) + \mathscr{H}_I(p_I, \partial_{p_I}))\,\Phi_1 = \partial_\tau \Phi_0 - \mathscr{H}_x(x, \partial_x)\Phi_0. \tag{S.29}$$

We now proceed to solve Eqs. (S.28) and (S.29) order by order. Regarding $\Phi_0(x, p_A, p_I, \tau)$, by inspection of Eq. (S.28), we assume $\Phi_0(x, p_A, p_I, \tau)$ to have the form $\Phi_0(x, p_A, p_I, \tau) = \phi_x(x, \tau)\phi_A(p_A|x)\phi_I(p_I|x)$. In what follows, to simplify the notation, we will omit the *conditioned to x* when we refer to ϕ_A and ϕ_I. Then Eq. (S.28) becomes:

$$\phi_x(x,\tau)\left(\phi_I(p_I)\mathscr{H}_A(p_A, \partial_{p_A})\phi_A(p_A) + \phi_A(p_A)\mathscr{H}_I(p_I, \partial_{p_I})\phi_I(p_I)\right) = 0,$$

which implies that ϕ_A and ϕ_I satisfy:

$$\mathscr{H}_A(p_A, \partial_{p_A})\phi_A(p_A) = 0,$$
$$\mathscr{H}_I(p_A, \partial_{p_I})\phi_I(p_I) = 0.$$

Explicit solutions to these equations are provided by the Wentzel-Kramers-Brillouin (WKB) approximation, which holds when $\Omega_S >> 1$. Such approximations for non-linear birth-and-death processes have been thoroughly studied in the literature (30, 31). To the lowest order, ϕ_A and ϕ_I are given by:

$$\phi_J = \frac{\mathscr{N}_J}{\sqrt{B_J(p_J,x)D_J(p_J,x)}} \exp\left(-\Omega_S \int_0^{p_J} \log\left(\frac{D_J(s,x)}{B_J(s,x)}\right) ds\right), \tag{S.30}$$

$\mathscr{N}_A$ and $\mathscr{N}_I$ being normalisation constants so that $\int_0^{\rho_J} \phi_J dp_J = 1$ with $J = A, I$.

Equation (S.28) provides no information on $\phi_x(x,\tau)$ and thus we need to resort to the first order Eq. (S.29), in order to determine $\phi_x(x,\tau)$. When tackling Eq. (S.29), we find a well-known problem in singularly perturbed problems. Since $(\mathscr{H}_A(p_A, \partial_{p_A}) + \mathscr{H}_I(p_I, \partial_{p_I}))\, f = 0$ has non-trivial solutions, for the corresponding inhomogeneous problem Eq. (S.29) to have solution(s), its right hand side needs to satisfy additional conditions (32). Such conditions are known as solvability conditions and they eventually provide the equation for determining the for of ϕ_x (29, 32–34). Consider $\varphi(p_A, p_I)$ defined as:

$$\varphi(p_A, p_I) = \begin{cases} 1 \text{ if } (p_A, p_I) \in \mathscr{D}, \\ 0 \text{ otherwise,} \end{cases}$$

where $\mathscr{D} = [0, \rho_A] \times [0, \rho_I]$, and consider also its scalar product with the left hand side of Eq. (S.29):

$$\langle \varphi, (\mathscr{H}_A(p_A, \partial_{p_A}) + \mathscr{H}_I(p_I, \partial_{p_I}))\,\Phi_1 \rangle = \langle {\mathscr{H}^\dagger}_A(p_A, \partial_{p_A})\varphi + \mathscr{H}_I^\dagger(p_I, \partial_{p_I})\varphi, \Phi_1 \rangle = 0,$$

which follows in a straightforward way from the form of the operators $\mathscr{H}_A$ and $\mathscr{H}_I$. The scalar product is defined by: $\langle \varphi, \phi \rangle = \int_{\mathscr{D}} \varphi\phi\, dp_A dp_I$. The solvability condition thus reads:

$$0 = \int_{\mathscr{D}} \partial_\tau \Phi_0 dp_A dp_I - \int_{\mathscr{D}} \mathscr{H}_x(x, \partial_x)\Phi_0 dp_A dp_I.$$

Taking into account that $\Phi_0(x, p_A, p_I, \tau) = \phi_x(x,\tau)\phi_A(p_A)\phi_I(p_I)$, this condition leads to:

$$\partial_\tau \phi_x = \langle \mathscr{H}_x(x, \partial_x) \rangle \phi_x \tag{S.31}$$

where

$$\langle \mathscr{H}_x(x, \partial_x) \rangle \phi_x = \int_{\mathscr{D}} \left(\Omega_T \left(\left(e^{\partial_x} - 1\right) B_x(x)\phi_x + \left(e^{-\partial_x} - 1\right) D_x(x)\phi_x\right)\right) \phi_A \phi_I dp_A dp_I.$$

In order to provide an analytic expression of the averaged operator $\langle \mathscr{H}_x \rangle$, we recall the WKB approximation of ϕ_A and ϕ_I, Eq. (S.30). Their exponential form and the fact that we are considering the situation where $\Omega_S >> 1$ (with $\Omega_T >> \Omega_S$) allows us to obtain an asymptotic approximation of the integrals involved in $\langle B_x \rangle$ and $\langle D_x \rangle$ using Laplace's method (35). Under such approximation, to the lowest order (36), the averaged operator $\langle \mathscr{H}_x \rangle$ is given by:

$$\langle \mathscr{H}_x(x, \partial_x) \rangle \phi_x = \Omega_T \left(\left(e^{\partial_x} - 1\right) \langle B_x \rangle(x)\phi_x + \left(e^{-\partial_x} - 1\right) \langle D_x \rangle(x)\phi_x\right),$$

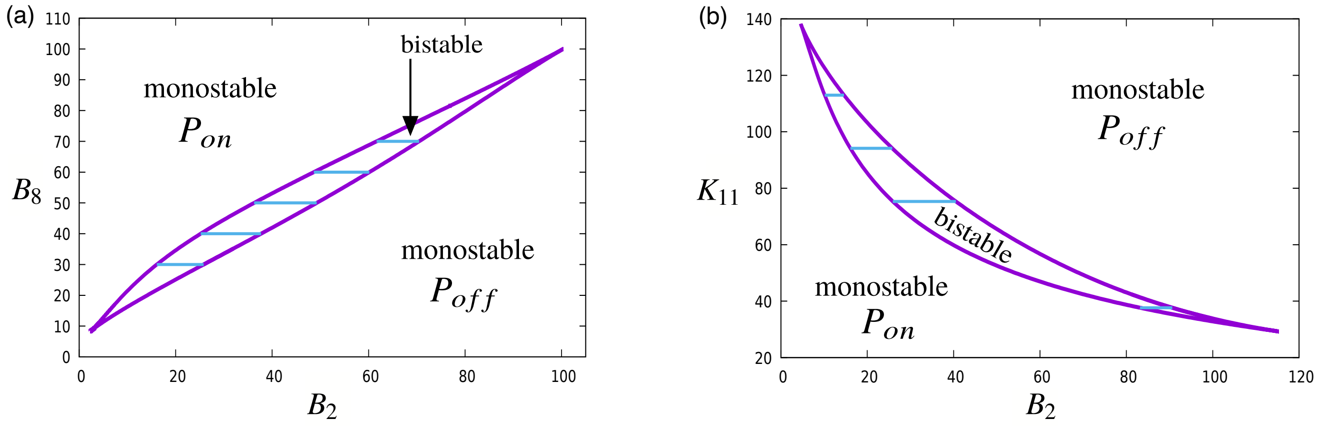

Figure A: **Two-parametric bifurcation diagrams showing the curves of saddle-node bifurcations of system Eqs. (1-3) from the main text.** (a) Parameter plane (B_2, B_8), with $K_{11} = 94.155549$. (b) Parameter plane (B_2, K_{11}), with $B_8 = 30$. The violet solid curve represents the parameter values at which a saddle-node bifurcation occurs. The inner blue lines are specific values of B_8 for which we have computed the relative size, P_{off}, of the basin of attraction of the OFF state (see Fig. S2 below).

where

$$\langle B_x \rangle(x) = \rho \left[1 + (C_1 + C_4 q_I^*(x)) \frac{q_A^*(x)x}{\lambda_x q_I^*(x) + q_A^*(x)x} \right],$$

and

$$\langle D_x \rangle(x) = \rho \left[C_2 x + C_4 q_I^*(x) \frac{q_A^*(x)x}{\lambda_x q_I^*(x) + q_A^*(x)x} \right].$$

$q_A^*(x)$ and $q_I^*(x)$ are defined as:

$$q_A^*(x) = \max_{p_A} \mathscr{S}_A(p_A, x) \ \text{ and } \ q_I^*(x) = \max_{p_I} \mathscr{S}_I(p_I, x), \tag{S.32}$$

with $\mathscr{S}_J(p_J, x) = \int_0^{p_J} \log \frac{D_J(s,x)}{B_J(s,x)} ds$, $J = A, I$. Note that $q_A^*(x)$ and $q_I^*(x)$ are the roots of the corresponding growth rates $G_J(p_J, x) = B_J(p_J, x) - D_J(p_J, x) = 0$, and therefore gives you the same as looking for the stationary points of the mean-field equations. Recall that we are choosing parameter values (in particular, $\tilde{\rho}_A$, $\tilde{\rho}_I$, $\tilde{\rho}_{p_A}$, and $\tilde{\rho}_{p_I}$) so that $G_J(p_J, x) = 0$ has only one solution in the interval $[0, \rho_J]$.

At this point we can calculate the analytic expression of the steady state PDF $\phi_x(x)$ by resorting again to the WKB approximation of the steady state averaged Master Equation, Eq. (S.31):

$$\langle \mathscr{H}_x(x, \partial_x) \rangle \phi_x(x) = 0$$

which is given by (31)

$$\phi_x = \frac{\mathscr{N}_x}{\sqrt{\langle B_x \rangle(x) \langle D_x \rangle(x)}} \exp\left(-\Omega_T \mathscr{S}(s) ds\right) \tag{S.33}$$

where

$$\mathscr{S}(s) = \int_0^x \log \frac{\langle D_x \rangle(s)}{\langle B_x \rangle(s)} ds.$$

S.1.3 Deterministic model: Basins of attraction and bistability

In order to understand the dependency of the deterministic model given by Eqs. (1)-(3) to parameters as well as the effect of the initial amount of transcription factor (TF) in the achievement of the *on* or the *off* states, we have explored two 2-parametric sections in the 3-parametric space (B_2, B_8, K_{11}), both included in the bistability region, namely $K_{11} = 94.155549$ and $B_8 = 30$. For each section, we first determined the boundaries of the bistability region by using numerical continuation methods from XPP/AUTO (37), see Fig. A, violet solid curves. The bifurcation diagram displays the curve of saddle-node bifurcations: if we

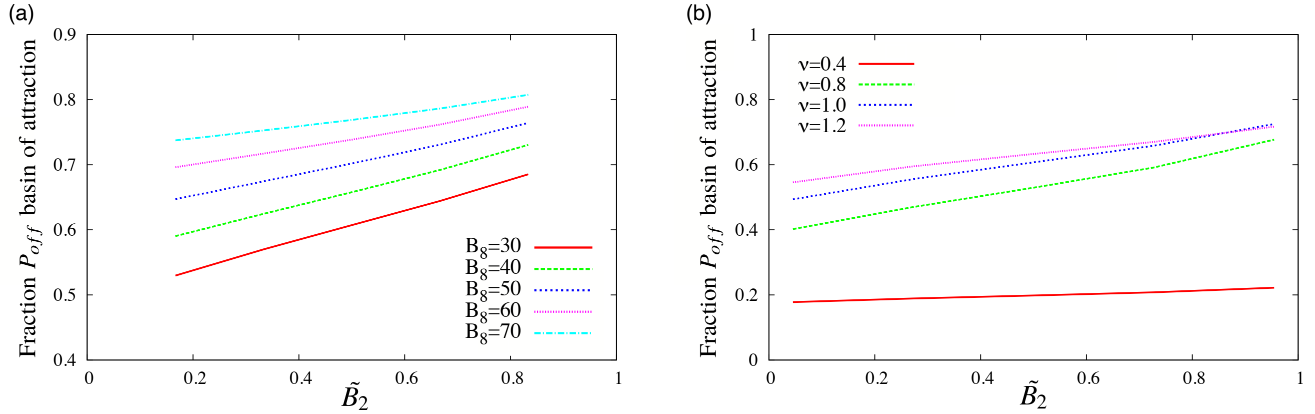

Figure B: **Relative size of the basin of attraction of P_{off} for parameter sets within the bistability region.** Note that the size of the basin of attraction for $P_{on} = 1 - P_{off}$. (a) One-dimensional representation in terms of $\widetilde{B}_2$ for 5 different values of B_8, with $K_{11} = 94.155549$. (b) One-dimensional representation in terms of $\widetilde{B}_2$ for 4 different values of K_{11}, with $B_8 = 30$. As defined in (S.34), $\widetilde{B}_2$ stands for a normalisation of parameter B_2.

let only one free parameter (say, B_2) and take the other parameters fixed (B_8 and K_{11} in this case), two saddle-node bifurcations arise. Beyond both bifurcation values, the system is monostable; at the first bifurcation in the direction of increasing B_2, two equilibria appear (one stable and another unstable) and the system becomes bistable (two stable equilibria) while, at the second bifurcation, two equilibria disappear leading to a monostable system again. More specifically, the TF is at the ON state above the bistability region in the space (B_2, B_8), and reaches the OFF state below it (panel (a) in Fig. A). The situation is reversed in the parameter space (B_2, K_{11}) (see panel (b) in Fig. A).

To estimate the relative sizes of the basins of attraction in the bistability regimes we fix different values of B_8 and increase parameter B_2 (see Fig. A, horizontal dotted lines). As mentioned, the two possible equilibrium states, P_{off} and P_{on}, can be achieved depending on the initial conditions of variables x, q_A, and q_I. For each specific choice of the parameter values, we are interested in quantifying how likely a given initial number of TF molecules, of H3K4me3, and H3K27me3 make the system to achieve the *on* or *off* state. To do so, we have simulated 24×10^6 initial conditions regularly sampled within the cube $[0.005, 3.00] \times [0.005, 1.00] \times [0.005, 1.00]$ in the (x, q_A, q_I)-space to determine the fraction of initial conditions that reach P_{off} (alternatively, $P_{on} = 1 - P_{off}$). We take this fraction as an approximation of the size of the basin of attraction of each steady state. For each initial condition we have numerically solved Eqs. (1-3) in the Main Manuscript with a fourth-order Runge-Kutta method (with a constant time step-size $\delta t = 10^{-2}$). The stabilisation of the solutions towards a given fixed point has been established when the Euclidean norm of the vector field, $||\mathscr{F}||_2 := |\sqrt{\dot{x}^2 + \dot{q_A}^2 + \dot{q_I}^2}|$, satisfies $||\mathscr{F}||_2 < 10^{-12}$.

Figure B shows the relative size of the basin of attraction of P_{off} for parameter sets within the bistability region. As in Fig. A, we study both the case $K_{11} = 94.155549$ and the case $B_8 = 30$ (see panels (a) and (b), respectively). For the sake of comparison, for each B_8 or K_{11} value, we have normalized the bistability interval to be of length 1 by using the information about the two saddle-node bifurcations that bound the bistability region. We define a parameter

$$\widetilde{B}_2 := \frac{B_2 - B_{2,sn1}}{\Delta B_2}, \qquad \text{(S.34)}$$

where $B_{2,sn1}$ (resp., $B_{2,sn2}$) is the value of B_2 at the lowest saddle-node bifurcation and $\Delta B_2 = B_{2,sn2} - B_{2,sn1}$ is the bistability interval length. In particular, for $K_{11} = 94.155549$ and $B_8 = 30, 40, 50, 60, 70$, $B_{2,sn1} \approx 16.0847, 25.1832, 36.2579, 48.6356, 61.6569$, respectively, and $\Delta B_2 \approx 9.7056, 12.5102, 12.9733, 11.5153, 8.7999$, respectively. For $B_8 = 30$ and $K_{11} = 94.155549\,\nu$, for $\nu = 0.4, 0.8, 1.0, 1.2$, we have that $B_{2,sn1} \approx 83.1726, 25.9152, 16.0847, 10.1504$, respectively, and $\Delta B_2 \approx 7.4610, 14.6055, 9.7056, 4.4709$, respectively. In the main text we show interpolated surfaces of the values computed with this procedure (see Fig. 6 in the main manuscript).

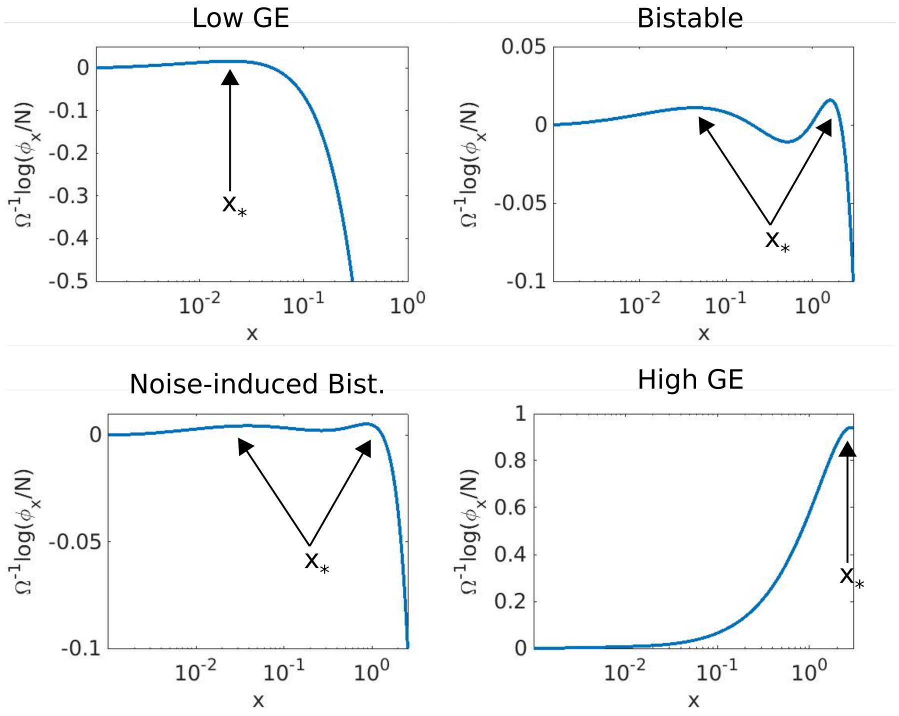

Figure C: $\phi_x(x)$ **for different sets of parameter values representative of each sub-ensemble**. For each case, we mark the corresponding value(s) of x_*.

S.1.4 Parameter sensitivity analysis: Ensemble approach

S.1.4.1 Ensemble description

The ensembles invoked in the Results section of the main text have been generated by a uniform sampling of a 16-dimensional parameter space. Each parameter set is thus described by a vector, θ, in such space, whose components are:

$$\theta = (A_1, A_3, B_2, K_2, K_5, K_6, \lambda_A, \lambda_x, A_7, A_9, B_8, K_8, K_9, K_{11}, K_{12}, \lambda_I)$$

(see Table B). Each component of θ is independently generated by sampling from a uniform prior distribution within a certain prescribed range. The three different ensembles considered in this paper correspond to changes in the admissible range in (some of) the parameter values, as described in Table C

Table C: Range of variation of each parameter within each of the ensembles.

A_1	A_3	B_2	K_2	K_5	K_6	λ_A	λ_x
[0.01,10]	[0.001,0.1]	[0.001,100]	[0.01,400]	[0.01,400]	[0.1,10]	[0.1,10]	[0.1,10]
A_7	A_9	B_8	K_8	K_9	K_{11}	K_{12}	λ_I
[0.01,10]	[0.001,1]	[0.001,100]	[0.01,200]	[0.01,200]	[0.01,400]	[0.1,10]	[0.1,10]

S.1.4.2 Classification into sub-ensembles

As described in Section S.1.4.1, each parameter set is generated at random by sampling each component (parameter) independently from its corresponding prior distribution. Parameter values thus generated are then classified into three sub-ensembles (see Fig. 2(a) of the main text):

1. **Viability**. A randomly generated parameter set, θ, is first checked for viability regarding the uniqueness of the solution of the variational problem from Eq. (S.32). The solution of such problem corresponds to finding the positive real roots

of a 4th degree polynomial. If the number of such roots is equal to one for all values of x, then θ is deemed as viable, otherwise it is rejected.

2. **Monostable**. Viable parameter sets such that both $\partial_x \mathscr{S}(x) = 0$ and $\partial_x \mathscr{U}(x, \Omega_T) = 0$ have one and only one solution are classified as monostable. Such parameter sets produce a monomodal steady state PDF of the gene expression level, x. Monostable parameter sets are themeselves classified into two subclasses:

    - **High Gene Expression**. If θ is such that the mode of $\phi_x(x)$, $x_*(\theta)$ (defined as the unique root of $\partial_x \mathscr{U}(x, \Omega_T) = 0$, see Fig. C and Fig. 3 of the main text), satisfies $x_*(\theta) \geq x_{THR}$, θ is classified as belonging to the High Gene Expression (High GE) sub-ensemble.
    - **Low Gene Expression**. If $x_*(\theta) < x_{THR}$, θ is classified as belonging to the Low Gene Expression (Low GE) sub-ensemble.

3. **Bistable**. Viable parameter sets such that both $\partial_x \mathscr{S}(x) = 0$ and $\partial_x \mathscr{U}(x, \Omega_T) = 0$ have three positive solutions are classified as bistable.

4. **Noise-Induced Bistable**. Viable parameter sets such that both $\partial_x \mathscr{S}(x) = 0$ have one and only one positive solution and $\partial_x \mathscr{U}(x, \Omega_T) = 0$ have three positive solutions are classified as noise-induced bistable (N.-I. Bistable).

Table D: Brief summary of the generated ensemble of θs.

Total Number (Viable)	High GE (%)	Low GE (%)	Bistable (%)	Noise-Induced Bist. (%)
19576	21.026	73.774	4.771	0.429

In Table D we present a brief summary of the results obtained for our ensemble (Section S.1.4.1). Details of the (sub)ensemble generated are provided in Fig.2(a)-(c) and Fig. 6(a)-(b) in the main text and Fig. D, where we show the distributions (boxplots) for each parameter (i.e. each component of the vector θ). We will use this data to detect which parameters show statistically significant differences between sub-ensembles to determine which of them are key to observe the behaviour associated with each sub-ensemble (high/low GE, bistability, noise-induced bistability) (7, 8).

Analysis of statistically significant differences between sub-ensembles. We start our analysis by comparing the marginal posteriors PDFs corresponding to the different sub-ensembles (see Figs. D). The rationale for performing this analysis is as follows (see (7, 8) for more details). The classification of parameter sets into sub-ensembles corresponds to physical/biological different behaviours. We expect that by discerning which parameters present statistically significant differences between sub-ensembles, we can identify which of them are key to produce the behaviour associated to a particular sub-ensemble (7, 8). For example, consider the marginal CDFs for the parameter A_3. By inspection, the marginal CDFs corresponding to the bistable sub-ensembles are clearly different from those corresponding to the monostable sub-ensembles (high and low GE). Furthermore, the data shows a clear bias towards lower values of A_3 within the bistable sub-ensembles. These results suggest that A_3 is one of the key parameters involved in bistable behaviour and that, if we wish to drive a bistable-to-monostable transition changing A_3, a feasible strategy consists of increasing the value of A_3 (7, 8). Here, we perform this analysis in a systematic and quantitative way.

In order to perform such comparison, we use the two-sample Kolmogorov-Smirnov (KS) test. Such test returns a decision regarding the null hypothesis that both data sets are drawn from the same continuous distribution. The result is $h = 1$ if the test rejects the null hypothesis at the prescribed significance level, and $h = 0$ otherwise. The results are summarised in Table E.

We first observe that the division of the monostable sub-ensemble into two sub-ensembles (high and low GE) is statistically meaningful. In Table E(a), where we present the summary of the comparison between the marginal CDFs for the high-GE and low-GE sub-ensembles, we see that the marginal distributions for all parameters exhibit statistically significant differences. Only for K_8 the statitiscal evidence for such difference is weaker, as shown by the corresponding p-values. In fact, for Ensemble 2 the KS test cannot reject the null hypothesis.

We now proceed to compare the bistable sub-ensemble with the high GE and low GE sub-ensembles. The results are shown in Table E(b)-(c). By simple visual inspection, we can tell that the marginal posteriors of the bistable sub-ensemble share more similarities with the High GE sub-ensemble than with the low GE sub-ensemble. Specifically, the KS test cannot reject the null hypothesis for only two (K_9 and λ_I) out of the sixteen parameters. Furthermore, for (A_1, K_5, and K_{12}) the rejection of the null

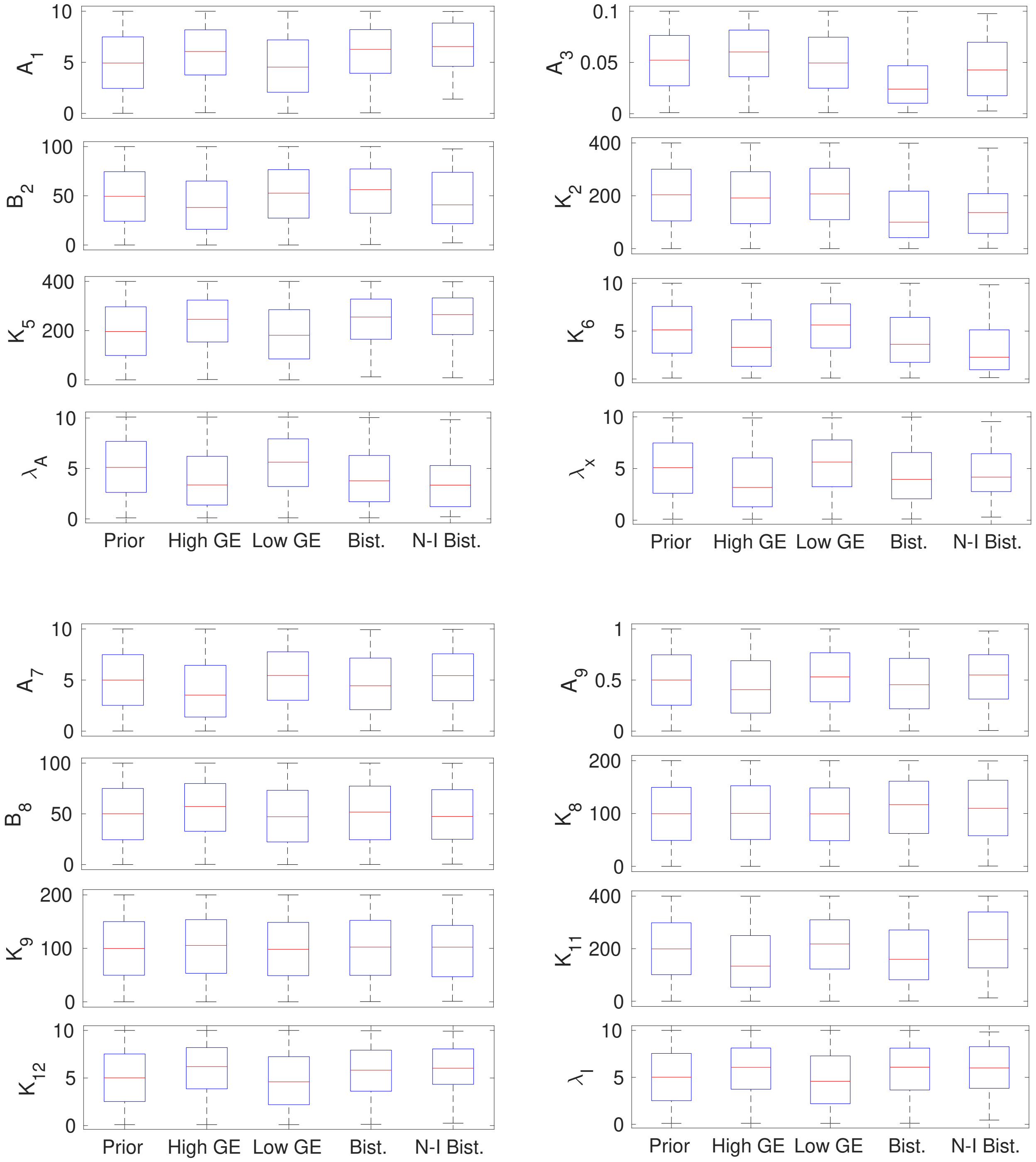

Figure D: **Boxplots for the generated distribution of parameters.** Each plot shows the boxplot corresponding to a specific parameter (see Table B) within each sub-ensemble. For reference, we also show the boxplot for the prior distribution. The upper (lower) panel shows results for parameters related to the modifiers of H3K4 (H3K27).

Table E: Significance level $\alpha = 0.075$.

(a) High vs Low GE

	h	p-value
A_1	1	5.454601e-90
A_3	1	5.870815e-46
B_2	1	6.103946e-64
K_2	1	3.503613e-06
K_5	1	1.669834e-96
K_6	1	1.056691e-166
λ_A	1	1.282697e-155
λ_x	1	1.885551e-188
A_7	1	1.732416e-115
A_9	1	2.317481e-53
B_8	1	1.130947e-37
K_8	1	5.041977e-02
K_9	1	1.262770e-05
K_{11}	1	3.614244e-143
K_{12}	1	1.582725e-90
λ_I	1	1.816639e-86

(b) High GE vs Bistable

	h	p-value
A_1	1	7.471566e-02
A_3	1	7.804065e-110
B_2	1	4.189324e-27
K_2	1	1.408325e-38
K_5	1	6.347346e-02
K_6	1	1.179497e-03
λ_A	1	1.092038e-03
λ_x	1	8.777037e-11
A_7	1	9.895137e-08
A_9	1	3.844402e-03
B_8	1	2.351621e-05
K_8	1	6.853621e-06
K_9	0	1.786971e-01
K_{11}	1	3.094784e-08
K_{12}	1	1.587451e-02
λ_I	0	6.137966e-01

(c) Low GE vs Bistable

	h	p-value
A_1	1	1.962069e-31
A_3	1	1.417666e-64
B_2	1	8.579826e-04
K_2	1	9.754648e-60
K_5	1	2.332903e-37
K_6	1	5.720654e-38
λ_A	1	3.098753e-31
λ_x	1	5.410590e-25
A_7	1	4.027444e-10
A_9	1	2.025408e-06
B_8	1	1.832587e-02
K_8	1	2.607908e-07
K_9	0	5.258169e-01
K_{11}	1	1.212437e-22
K_{12}	1	1.952996e-20
λ_I	1	1.002306e-21

(d) Bistable vs Noise-induced Bimodal

	h	p-value
A_1	0	2.897352e-01
A_3	1	3.662555e-04
B_2	1	1.608322e-03
K_2	0	1.115658e-01
K_5	0	6.618277e-01
K_6	1	8.472578e-03
λ_A	0	2.219046e-01
λ_x	0	9.567488e-02
A_7	1	6.993023e-02
A_9	0	1.994637e-01
B_8	0	9.308129e-01
K_8	0	9.117183e-01
K_9	0	7.579813e-01
K_{11}	1	4.657800e-03
K_{12}	0	2.781216e-01
λ_I	0	6.847780e-01

hypothesis is supported by weaker statistical evidence (see Table E(b)). For the low GE sub-ensemble, the number of such parameters is reduced to three (data not shown).

Further analysis is carried out by comparing the bistable sub-ensembles (regular and noise-induced) as shown in Table E(d). Such an analysis produces the result that only 5 parameters exhibit statistically significant differences regarding their marginal posteriors. Parameters B_2 and K_{11} are specially relevant cases within such subset of parameters, as they are closely related to ER enzyme activity and thus to the levels of positive and negative epigenetic marks, respectively.

S.1.5 Ensemble averages

Recall that the steady state PDF $\phi_x(x)$, as given by Eq. (S.35):

$$\phi_x(x|\theta) = \frac{\mathscr{N}_x}{\sqrt{\langle B_x\rangle(x)\langle D_x\rangle(x)}}\exp\left(-\Omega_T \mathscr{S}(x)\right) \tag{S.35}$$

where

$$\mathscr{S}(x) = \int_0^x \log\frac{\langle D_x\rangle(s)}{\langle B_x\rangle(s)}ds.$$

We have also made explicit in the notation that the steady-state PDF ϕ_x is conditioned to the choice of a particular set of parameter values, θ.

In order to calculate ensemble averages of positive and negative marks,

$$\langle q_A|\theta\rangle_x = \int_0^\infty q_A(x)\phi_x(x|\theta)dx$$

and

$$\langle q_I|\theta\rangle_x = \int_0^\infty q_I(x)\phi_x(x|\theta)dx,$$

respectively, we will need to approximate the value of the corresponding integrals, since in general we can give no closed exact expression. Since we are assuming that $\Omega_T \gg 1$, we apply the Laplace method to approximate such integrals. Such method applies to integrals of the type:

$$I(\Omega_T) = \int_a^b f(x)e^{-\Omega_T g(x)}dx.$$

In the limit of $\Omega_T \gg 1$, if $g(x)$ exhibits a (unique) minimum at $x_* \in (a,b)$, the integral can be approximated by (35):

$$I(\Omega_T) \approx \sqrt{\frac{2\pi}{\Omega_T g''(x_*)}} f(x_*) e^{-\Omega_T g(x_*)}.$$

If $g(x)$ has two minima within (a,b), $x_{*_-} \in (a, x_{*_0})$ and $x_{*_+} \in (x_{*_0}, b)$, where x_{*_0} is the maximum of $g(x)$, we simply write:

$$I(\Omega_T) = \int_a^{x_{*_0}} f(x) e^{-\Omega_T g(x)} dx + \int_{x_{*_0}}^{b} f(x) e^{-\Omega_T g(x)} dx$$

so that the integral can now be approximated by:

$$I(\Omega_T) \approx \sqrt{\frac{2\pi}{\Omega_T g''(x_{*_-})}} f(x_{*_-}) e^{-\Omega_T g(x_{*_-})} + \sqrt{\frac{2\pi}{\Omega_T g''(x_{*_+})}} f(x_{*_+}) e^{-\Omega_T g(x_{*_+})}.$$

In our case, we have that $f(x) \equiv \frac{\mathscr{N}_x}{\sqrt{\langle B_x \rangle \langle D_x \rangle}} q_J(x)$, where $J = A, I$, $g(x) = \mathscr{S}(x)$, and

$$g'' = \mathscr{S}'' = \frac{\langle D_x \rangle'}{\langle D_x \rangle} - \frac{\langle B_x \rangle'}{\langle B_x \rangle},$$

where

$$\langle B_x \rangle' \equiv \frac{d\langle B_x \rangle}{dx} = \partial_x \langle B_x \rangle - \left.\frac{\partial_x G_A}{\partial_{p_A} G_A}\right|_{p_A = q_A^*} \partial_{p_A} \langle B_x \rangle - \left.\frac{\partial_x G_I}{\partial_{p_I} G_I}\right|_{p_I = q_I^*} \partial_{p_I} \langle B_x \rangle$$

and

$$\langle D_x \rangle' \equiv \frac{d\langle D_x \rangle}{dx} = \partial_x \langle D_x \rangle - \left.\frac{\partial_x G_A}{\partial_{p_A} G_A}\right|_{p_A = q_A^*} \partial_{p_A} \langle D_x \rangle - \left.\frac{\partial_x G_I}{\partial_{p_I} G_I}\right|_{p_I = q_I^*} \partial_{p_I} \langle D_x \rangle.$$

Note that, since we cannot give expressions for $q_A^*(x)$ and $q_I^*(x)$ in closed analytical form, we have used the implicit function theorem and the fact that $q_J^*(x)$ satisfies $G_J(x, q_J^*) = B_J(x, q_J^*) - D_J(x, q_J^*) = 0$ $(J = A, I)$.

Using these results, we can compute the ensemble-averaged positive and negative marks for both the monostable sub-ensembles (Low GE and High GE) and the bistable sub-ensemble

$$\langle q_A \rangle_H = \frac{1}{N_H} \sum_{\theta \in HGSE} \langle q_A | \theta \rangle_x, \; \langle q_I \rangle_H = \frac{1}{N_H} \sum_{\theta \in HGSE} \langle q_I | \theta \rangle_x, \tag{S.36}$$

$$\langle q_A \rangle_L = \frac{1}{N_L} \sum_{\theta \in LGSE} \langle q_A | \theta \rangle_x, \; \langle q_I \rangle_L = \frac{1}{N_L} \sum_{\theta \in LGSE} \langle q_I | \theta \rangle_x, \tag{S.37}$$

$$\langle q_A \rangle_B = \frac{1}{N_B} \sum_{\theta \in BSE} \langle q_A | \theta \rangle_x, \; \langle q_I \rangle_B = \frac{1}{N_B} \sum_{\theta \in BSE} \langle q_I | \theta \rangle_x, \tag{S.38}$$

where the subindexes H, L, and B refer to average with respect to the High GE ($HGSE$), Low GE ($LGSE$), and bistable (BSE) sub-ensembles, respectively. N_H, N_L, and N_B are the cardinals of the High GE, Low GE, and bistable sub-ensembles, respectively.

S.2 SUPPLEMENTARY TABLES AND FIGURES

REFERENCE

1. Keener, J., and J. Sneyd, 1998. Mathematical physiology. Springer-Verlag, New York, NY, USA.

2. Ingalls, B. P., 2013. Mathematical modelling in systems biology. An introduction. The MIT Press, Cambridge, Massachusets, USA.

3. Dodd, I. B., M. A. Micheelsen, K. Sneppen, and G. Thon, 2007. Theoretical analysis of epigenetic cell memory by nucleosome modification. *Cell* 129:813–822.

4. Sneppen, K., M. A. Michelson, and I. B. Dodd, 2008. Ultrasensitive gene regulation by positive feedback loops in nucleosome modification. *Mol Syst Biol.* 4:182.

Table F: Parameter set 1 (Monostable Low GE with A_3 min). $\rho_A = 1$, $\rho_I = 1$. $\varepsilon_1 = 0.1$

Methylation/demethylation of H3K4
$A_1 = 4.795239$, $A_3 = 0.001006$, $B_2 = 64.091515$, $K_2 = 171.650858$, $K_5 = 169.260923$
$K_6 = 4.256390$, $\lambda_A = 8.658754$
Methylation/demethylation of H3K27
$A_7 = 7.110925$, $A_9 = 0.726804$, $B_8 = 89.408867$, $K_8 = 95.702027$, $K_9 = 28.886043$
$K_{11} = 279.510584$, $K_{12} = 8.484769$, $\lambda_I = 0.808614$
Gene regulatory network
$C_1 = 150$, $C_2 = 50$, $C_4 = 1000$, $\lambda_X = 7.504438$

Table G: Parameter set 2 (Bistability). $\rho_A = 1$, $\rho_I = 1$. $\varepsilon_1 = 0.1$

Methylation/demethylation of H3K4
$A_1 = 8.666099$, $A_3 = 0.040570$ $B_2 = 20.016911$, $K_2 = 271.229406$, $K_5 = 229.948007$
$K_6 = 6.207151$, $\lambda_A = 7.194034$
Methylation/demethylation of H3K27
$A_7 = 3.800899$, $A_9 = 0.063084$, $B_8 = 30.087626$, $K_8 = 88.410503$, $K_9 = 59.322552$
$K_{11} = 94.155549$, $K_{12} = 6.854684$, $\lambda_I = 1.310467$
Gene regulatory network
$C_1 = 150$, $C_2 = 50$, $C_4 = 1000$, $\lambda_X = 1.455620$

5. Menéndez, J. A., B. Corominas-Faja, E. Cuyàs, M. G. García, S. Fernández-Arroyo, A. F. Fernández, J. Joven, M. F. Fraga, and T. Alarcón, 2016. Oncometabolic nuclear reprogramming of cancer stemness. *Stem Cell Reports* 6:273–283.

6. Loubiere, V., A.-M. Martinez, and G. Cavalli, 2019. Cell fate and developmental regulation dynamics by polycomb proteins and 3D genome architecture. *BioEssays* 41:1800222.

7. Folguera-Blasco, N., E. Cuyàs, J. A. Menéndez, and T. Alarcón, 2018. Epigenetic regulation of cell fate reprogramming in aging and disease: A predictive computational model. *PLoS Comp. Biol.* 14:e1006052.

8. Folguera-Blasco, N., R. Pérez-Carrasco, E. Cuyás, J. A. Menéndez, and T. Alarcón, 2019. A multiscale model of epigenetic heterogeneity reveals the kinetic routes of pathological cell fate reprogramming. *PLoS Comp Biol.* 15:e1006592.

9. Thalheim, T., M. Herberg, M. Loeffler, and J. Galle, 2017. The Regulatory Capacity of Bivalent Genes– A Theoretical Approach. *Int J Mol Sci.* 18(5):1069.

10. Thalheim, T., L. Hopp, H. Binder, G. Aust, and J. Galle, 2018. On the Cooperation between Epigenetics and Transcription Factor Networks in the Specification of Tissue Stem Cells. *Epigenomes* 2:20.

11. Anderson, D. F., and T. G. Kurtz, 2010. Stochastic analysis of biochemical systems. Springer, New York, NY, USA.

12. Haseltine, E. L., and J. B. Rawlings, 2002. Approximate simulation of coupled fast and slow reactions for stochastic chemical kinetics. *J Chem Phys.* 117:6959–6969.

13. Rao, C. V., and A. P. Arkin, 2003. Stochastic chemical kinetics and the quasi-steady-state assumption: Application to the Gillespie algorithm. *J Chem Phys.* 118:4999–5010.

14. Cao, Y., D. T. Gillespie, and L. R. Petzold, 2005. Multiscale stochastic simulation algorithm with stochastic partial equilibrium assumption for chemically reacting systems. *J Comp Phys.* 206:395–411.

15. Cao, Y., D. T. Gillespie, and L. R. Petzold, 2005. The slow-scale stochastic simulation algorithm. *J Chem Phys.* 122:014116.

16. Goutsias, J., 2005. Quasiequilibrium approximation of fast reaction kinetics in stochastic biochemical systems. *J Chem Phys.* 122:184102.

Table H: Parameter set 3 (Noise-induced bistability). $\rho_A = 1$, $\rho_I = 1$. $\varepsilon_1 = 0.1$

Methylation/demethylation of H3K4
$A_1 = 6.563150$, $A_3 = 0.040022$, $B_2 = 19.965753$, $K_2 = 248.998412$, $K_5 = 290.668266$
$K_6 = 2.846470$, $\lambda_A = 1.419607$
Methylation/demethylation of H3K27
$A_7 = 4.389568$, $A_9 = 0.346774$, $B_8 = 63.121059$, $K_8 = 110.921072$, $K_9 = 20.259211$
$K_{11} = 330.633279$, $K_{12} = 9.438870$, $\lambda_I = 2.983005$
Gene regulatory network
$C_1 = 150$, $C_2 = 50$, $C_4 = 1000$, $\lambda_X = 5.607232$

Table I: Parameter set 4 (Monostable. High GE. A_3 min.). $\rho_A = 1$, $\rho_I = 1$. $\varepsilon_1 = 0.1$

Methylation/demethylation of H3K4
$A_1 = 3.720782$, $A_3 = 0.001024$, $B_2 = 7.810833$, $K_2 = 244.894942$, $K_5 = 169.260923$
$K_6 = 0.158431$, $\lambda_A = 5.960934$
Methylation/demethylation of H3K27
$A_7 = 6.595129$, $A_9 = 0.530692$, $B_8 = 66.009204$, $K_8 = 14.539354$ $K_9 = 155.153281$
$K_{11} = 122.173786$, $K_{12} = 9.148418$, $\lambda_I = 5.431558$
Gene regulatory network
$C_1 = 150$, $C_2 = 50$, $C_4 = 1000$, $\lambda_X = 1.701816$

17. Ball, K., T. G. Kurtz, L. Popovic, and G. Rempala, 2006. Asymptotic analysis of multi-scale approximations to reaction networks. *Ann App Prob.* 16:1925–1961.

18. E, W., D. Liu, and E. Vanden-Eijnden, 2007. Nested stochastic simulation algorithms for chemical kinetic systems with multiple time scales. *J Comp Phys.* 221:158–170.

19. Mastny, E. A., E. L. Haseltine, and J. B. Rawlings, 2007. Two classes of quasi-steady state model reductions for stochastic kinetics. *J Chem Phys.* 127:094106.

20. Kang, H.-W., and T. G. Kurtz, 2013. Separation of time-scales and model reduction for stochastic reaction networks. *Ann App Prob.* 23:529–583.

21. Alarcón, T., 2014. Stochastic quasi-steady state approximations for asymptotic solutions of the Chemical Master Equation. *J Chem Phys.* 140:184109.

22. Kang, H.-W., T. G. Kurtz, and L. Popovic, 2014. Central limit theorems and diffusion approximation for multiscale Markov chain models. *Ann App Prob.* 24:721–759.

23. de la Cruz, R., P. Guerrero, F. Spill, and T. Alarcón, 2015. The effects of intrinsic noise on the behaviour of bistable systems in quasi-steady state conditions. *J Chem Phys.* 143:074105.

24. Ibánez-Marcelo, E., and T. Alarcón, 2016. Surviving evolutionary escape on complex genotype-phenotype networks. *J Math Biol.* 72:623–647.

25. Ethier, S.N., and T. Kurtz, 1986. Markov processes: Characterization and Convergence. John Wiley & Sons, Inc.

26. de la Cruz, R., P. Guerrero, J. Calvo, and T. Alarcón, 2017. Coarse-graining and hybrid methods for efficient simulation of stochastic multi-scale models of tumour growth. *J Comp Phys.* 350:974–991.

27. Gardiner, C. W., 2009. Stochatic methods. Springer-Verlag, Berlin, Germany.

28. Horsthemke, W., and R. Lefever, 2006. Noise-induced transitions. Springer-Verlag, New York, NY, USA.

29. Bruna, M., S. J. Chapman, and M. J. Smith, 2014. Model reduction for slow-fast stochastic systems with metastable behaviour. *J Chem Phys.* 140:174107.

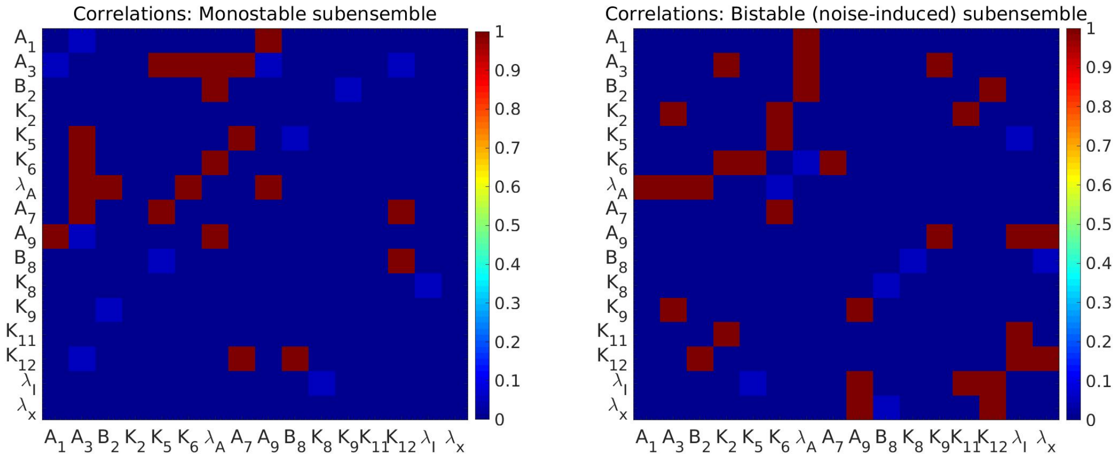

Figure E: **Correlations within the different sub-ensembles**. Red squares correspond to pairs such that the null hypothesis that the parameters are uncorrelated can be rejected (p-value < 0.05), dark blue, corresponds to p-values > 0.5 (uncorrelated pairs), and light blue, to p-values $\gtrsim$ 0.05, i.e. pairs of parameters for which the evidence of absence of correlations is weaker.

30. Hinch, R., and S. J. Chapman, 2005. Exponentially slow transitions on a Markov chain: the frequency of calcium sparks. *Eur J Appl Math.* 16:427–446.

31. Bressloff, P. C., 2014. Stochastic processes in cell biology. Springer-Verlag, Berlin, Germany.

32. Stakgold, I., 1998. Green's functions and boundary value problems. 2nd Ed. Wiley-Interscience, New York, NY, USA.

33. Ward, M. J., 1999. Exponential asymptotics and convection-reaction-diffusion models. *In* J. Cronin, and R. E. O'Mailley, editors, Analysing multiscale phenomena using singular perturbation methods. American Mathematical Society, Providence, RI, USA, 162–195.

34. Kogan, O., M. Khasin, B. Meerson, D. Schneider, and C. R. Myers, 2014. Two-strain competition in quasineutral stochastic disease dynamics. *Phys Rev E* 90:042149.

35. Murray, J. D., 1984. Asymptotic analysis. Springer-Verlag, New York, NY, USA.

36. Biancalani, T., and M. Assaf, 2015. Genetic toggle switch in the absence of cooperative binding: Exact results. *Phys Rev Lett.* 115:208101.

37. Ermentrout, B., 2002. Simulating, Analyzing, and Animating Dynamical Systems. Society for Industrial and Applied Mathematics. http://www.math.pitt.edu/~bard/xpp/xpp.html.

38. Sneeringer CJ, Scott MP, Kuntz KW, Knutson SK, Pollock RM, Richon VM, et al. Coordinated activities of wild-type plus mutant EZH2 drive tumour-associated hypertrimethylation of lysine 27 on histone H3 (H3K27) in human B-cell lymphomas. *Proc Natl Acad Sci U.S.A.* 2010; 107(49): 20980-20985.

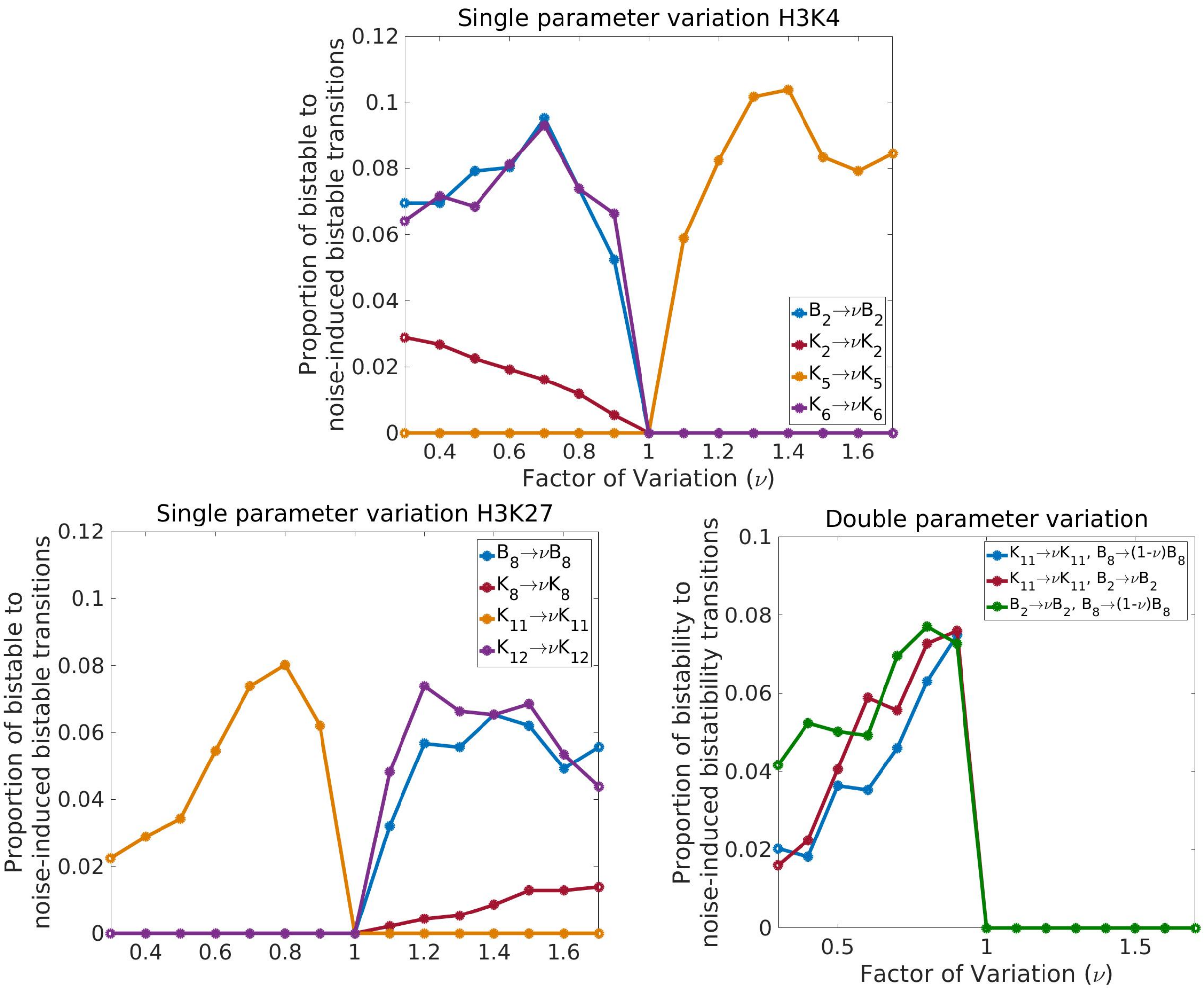

Figure F: **Further sensitivity analysis for the Bistable sub-ensemble.** These plots show our results regarding the sensitivity of the systems within the Bistable sub-ensemble to variations of selected parameters. Here we measure the probability that a system within the Bistable sub-ensemble moves to the Noise-Induced Bistable sub-ensmeble as a function of the factor of variation, ν.

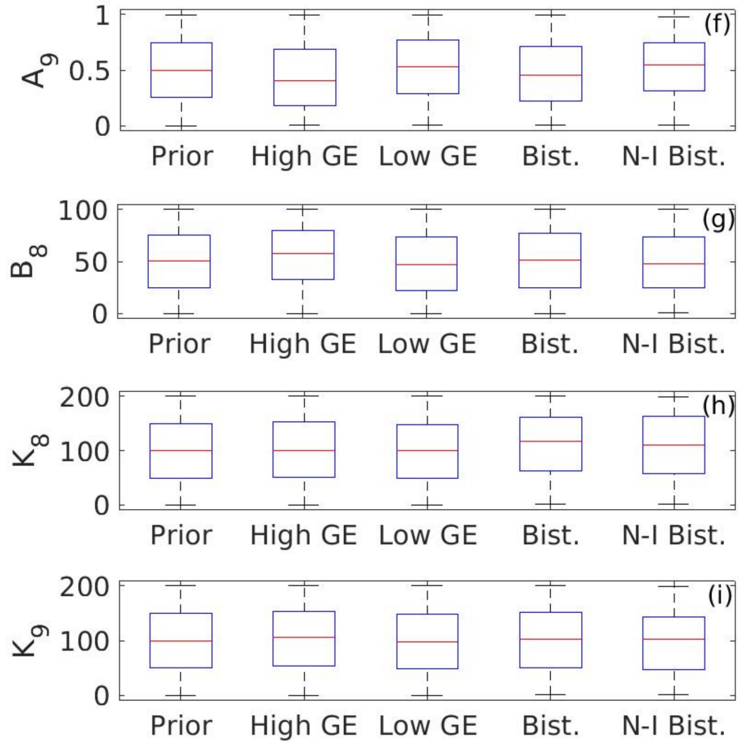

Figure G: **Statistics of specific parameters related to EZH2 enzymatic activity.** Quantitative analysis performed for four parameters related to the enzymatic kinetics of EZH2 (specifically K_M-like parameters, see Eq. (3) of the main text) for each of the four sub-ensembles shows no obvious differences between the Low GE PDFs and those corresponding to the High GE and the Bistable sub-ensembles. Statistical analysis shows significant differences, but with p-values very close to the significance level. This observation is consistent with direct *in vitro* measurements of the Michaelis-Menten constant, K_M, and the catalytic constant, k_{cat}, of the wild-type EZH2 and Y641F and Y641N mutants by Ref. (38), who found that in all variants of the enzyme, the K_Ms retained similar values within the error bars.
