# Supplementary material for: Bivalent chromatin as a therapeutic target in cancer: An in silico predictive approach for combining epigenetic drugs
Source: PLoS Comput Biol. 2021 Jun 21;17(6):e1008408. doi: 10.1371/journal.pcbi.1008408 (PMC8248646; doi:10.1371/journal.pcbi.1008408)
Supplement: S1 Table — Statistics regarding these quantities are shown in Figs 4 and 7. Recall that 〈f(x,z,θ)〉x=∫0∞f(x,z,θ)ϕx(x|θ)dx. (PDF) [file pcbi.1008408.s002.pdf]

S1 Table. Definition of the estimates or proxies for the activity of the chromatin modifiers. Statistics regarding these quantities are shown in Figs. **??** and **??**. Recall that $\langle f(x,z,\theta)\rangle_x = \int_0^\infty f(x,z,\theta)\phi_x(x|\theta)dx$.

	Definition	Description
Rate MLL2	$\mathcal{R}_{MLL2}(\theta) \equiv \max_q \langle B_A(x,q,\theta)\rangle_x$	Estimate (proxy) for the activity of MLL2 (rate of H3K4 trimethylation)
Rate KMD5	$\mathcal{R}_{KDM5}(\theta) \equiv \max_q \langle D_A(x,q,\theta)\rangle_x$	Estimate (proxy) for the activity of KDM5 (rate of H3K4me3 demethylation)
Rate EZH2	$\mathcal{R}_{EZH2}(\theta) \equiv \max_p \langle B_I(x,p,\theta)\rangle_x$	Estimate (proxy) for the activity of EZH2 (rate of H3K27 trimethylation)
Rate UTX	$\mathcal{R}_{UTX}(\theta) \equiv \max_p \langle D_I(x,p,\theta)\rangle_x$	Estimate (proxy) for the activity of UTX (rate of H3K4me3 demethylation)
